# The use of patient reported outcome measures in oncology clinical practice across Australia and New Zealand

**DOI:** 10.1186/s41687-023-00664-x

**Published:** 2024-01-02

**Authors:** Ashika D. Maharaj, Natasha Roberts, Michael Jefford, Jerome Ng, Claudia Rutherford, Bogda Koczwara

**Affiliations:** 1 Public Health and Preventative Medicine, Monash University, Clayton, VIC Australia; 2 The University of Queensland Centre for Clinical Research, Brisbane, Australia; 3 STARS Education and Research Alliance, Surgical Treatment and Rehabilitation Service (STARS), The University of Queensland and Metro North Health, Herston, QLD Australia; 4 Department of Health Services Research, Peter MacCallum Cancer Centre, Melbourne, VIC Australia; 5 Australian Cancer Survivorship Centre, Peter MacCallum Cancer Centre, Melbourne, VIC Australia; 6 Sir Peter MacCallum Department of Oncology, University of Melbourne, Melbourne, VIC Australia; 7 Te Whatu Ora Counties Manukau, Auckland, New Zealand; 8 School of Pharmacy, University of Auckland, Auckland, New Zealand; 9 Cancer Care Research Unit, Sydney Nursing School, University of Sydney, Sydney, NSW Australia; 10 The Daffodil Centre, The University of Sydney, a Joint Venture with Cancer Council NSW, Sydney, Australia; 11 Flinders Medical Centre and Flinders Health and Medical Research Institute, Flinders University, Adelaide, SA Australia; 12 Technology, Policy and Management, Delft University of Technology, Delft, The Netherlands

**Keywords:** Patient-reported outcome measures, Quality of care, Oncology, Health services research, Health policy

## Abstract

**Background:**

While there is increasing evidence on the benefits of PROMs in cancer care, the extent of routine collection and use of PROMs in clinical cancer practice across Australia and New Zealand (ANZ) is unknown. This study examined the prevalence and characteristics of PROMs use in routine clinical cancer care in ANZ.

**Methods:**

An online survey was designed and disseminated via professional societies and organisations using a snowball sampling approach to clinical and health administration professionals managing cancer care in ANZ. A poster advertising the study was also circulated on professional social media networks via LinkedIn and Twitter inviting health professionals from ANZ to participate if they were using or intending to use PROMs in clinical cancer practice. Responders opted into the survey via the survey link.

**Results:**

From 132 survey views, 91(response rate, 69%) respondents from 56 clinical practices across ANZ agreed to participate in the survey, and of these 55 (*n* = 55/91, 60%) respondents reported collecting PROMs within their clinical practice. The majority of the respondents were from the State of New South Wales in Australia (*n* = 21/55, 38%), hospital (*n* = 35/55, 64%), and a public setting (*n* = 46/55, 83%). PROMs were collected in all cancer types (*n* = 21/36, 58%), in all stages of the disease (*n* = 31/36, 86%), in an adult population (*n* = 33/36, 92%), applied in English (*n* = 33/36, 92%), and used to facilitate communication with other reasons (27/36, 75%). A geospatial map analysis provided insights into the variation in PROMs uptake between the two countries and in certain jurisdictions within Australia. This study also highlights the limited resources for PROMs implementation, and a lack of systematic priority driven approach.

**Conclusion:**

PROM use across Australia and New Zealand seems variable and occurring predominantly in larger metropolitan centres with limited standardisation of approach and implementation. A greater focus on equitable adoption of PROMs in diverse cancer care settings is urgently needed.

**Supplementary Information:**

The online version contains supplementary material available at 10.1186/s41687-023-00664-x.

## Background

A direct report from a patient on the status of their health condition without interpretation of their response by another individual is known as a patient-reported outcome (PRO) [1, 2] which is assessed with standardised questionnaires known as patient reported outcome measures (PROM). PROMs data have been used to inform health service performance and evaluation of costs, healthcare utilisation at the organisation level [3, 4]. The integration of a patients’ perspective is also important in informing areas for improvement within clinical practice and health care policy [5]. Over the past decade there has been growing interest in the use of PROMS in oncology clinical care as their routine collection has been associated with improved survival, reduced hospitalisations and emergency department presentations [6–9]. Further, with improved cancer treatments, the number of cancer survivors has increased [10, 11]. Positive findings from a systematic review by Graupner and colleagues recommend the use of PROMs in daily cancer care combined with feedback to the patients and relevant stakeholders [10] which may provide an important opportunity for a standardised approach to personalised cancer care [12].

While there is increasing evidence on the benefits of PROMs in cancer care, the extent of routine collection and use of PROMs in clinical cancer practice across Australia and New Zealand (ANZ) is unknown. For example, it is unclear whether the adoption of PROMs in clinical care varies, the perceived benefits of PROMs by healthcare personnel from oncology clinical practices; and how the data is used to inform individual patient care [13]. Addressing this gap will help inform the design of future health research and policies that may facilitate decision-making and encourage standardisation to the selection, implementation and evaluation of PROMs in the cancer clinical setting. This study examined the prevalence and characteristics of PROMs use in routine clinical care in ANZ such as how PROMs are collected, in which population and the infrastructure that supports the implementation of PROMs in the management of patients with cancer.

## Methods

### Study design

An online survey was designed and generated using the Qualtrics^©^ software, Version 2021 (https://www.qualtrics.com), tested with the study authors, and further piloted by the Clinical Oncology Society of Australia (COSA) PROMs working group for the validity and appropriateness of the survey questions (Additional file 1: Appendix 1).

### Survey development and testing

The questions in the survey were based on the items in the implementation guide for PROMs inclusion in clinical practice including the goals for collecting PROs, the patients, setting, and timing of assessments, questionnaire(s) in use, the mode for administering and scoring the questionnaire, processes for reporting results, clinic resources and to some extent evaluating the impact of the PRO intervention on the practice [14]. PROMs were defined as questionnaires that directly captured a patient’s perceptions on their disease or treatment related symptoms or side-effects, mental health concerns, or unmet needs. A ‘clinical practice’ was defined as any hospital, outpatient specialty clinic or community services. Responders who reported that they did not collect PROMs in clinical practice were asked if their clinical practice intended collecting PROMs in the future. The survey questions were either multichoice or short answer questions grouped in the following domains: (1) respondent demographics and clinical practice related information; (2) PROM collection of data; (3) resourcing, reporting and impact of PROMs data in clinical practice (Additional file 1: Appendix 1). A word document of the survey was sent for review to members from the COSA working group who provided feedback. Overall, members from the working group agreed that the content for the survey was appropriate and they shared further material from past surveys for the inclusion of additional questions. This survey was updated accordingly, developed online on Qualtrics and tested amongst the study authors. In addition, respondents were asked if they would be interested in taking part in further qualitative research.

### Respondents and recruitment

Clinical and health administration professionals who managed patients with cancer were recruited from public or private clinical practices from the different regions in Australia. New Zealand was classified as one region. Clinical settings included cancer centres (clinical practices within tertiary hospitals focused solely on the treatment of cancer) or comprehensive cancer centres (integrated cancer research, treatment and education centre), hospitals, or cancer-specific academic institutions, and oncology departments of general medical centres, hospitals or academic institutions which provided cancer care.

This study was endorsed by COSA [https://www.cosa.org.au/] and supported by the International Society for Quality of Life (ISOQOL) [https://www.isoqol.org/] to disseminate the survey to their members within ANZ. An invitation email with a participant information sheet that included the link to the survey was distributed via the aforementioned professional societies and organisations. Further, a snowball sampling approach was used where the members were asked to forward the invitation email to their colleagues or professional acquaintances.

A poster advertising the study was also circulated on professional social media networks via LinkedIn and Twitter (Additional file 2: Appendix 2).

Responders opted into the survey via the survey link and were asked to provide consent to participate before they could continue with the survey. Only respondents who collected PROMs within their clinical practice to guide patient care in cancer were eligible for the study. Respondents who did not collect PROMs or collected PROMs for research, clinical trials and/or quality improvement purposes were excluded from the final analysis.

### Analysis

Survey results were based on individual responses, and reported descriptively using frequencies, means/medians and percentages as appropriate. Data analyses were conducted in Microsoft Excel® Office 365 and Microsoft Power BI® 2022. A Chi Squared analysis was performed in Microsoft Excel® Office 365 to analyse the mean differences between responses of certain groups (collectors and non-collectors of PROMs in clinical practice for state, setting and organisational type). A two-sided *p* value of < 0.05 was considered statistically significant.

Open-ended questions were asked to determine the types of PROMs used in a respondent’s clinical practice, the process for developing PROMs if applicable, and whether the respondent was aware if the collection and reporting of PROMs had any impact on patient outcomes from their perspective. Where open-ended questions were used, responses were grouped into key themes or relevant areas and summarised quantitatively.

## Results

### Survey responses

**Fig. 1:**
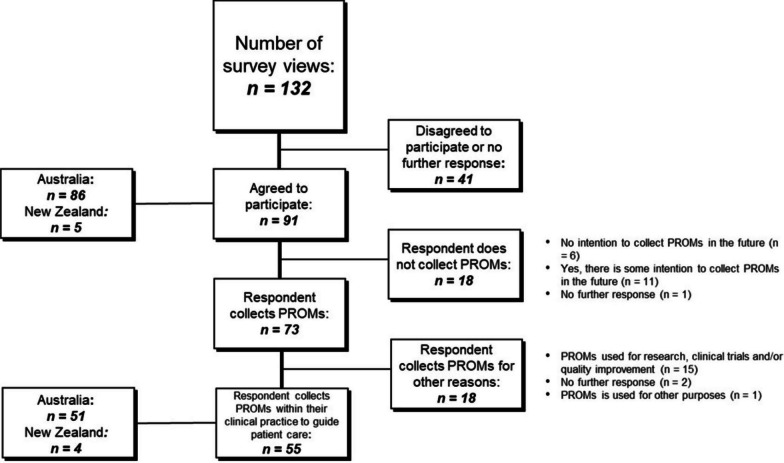
Overview of initial survey responses on the use of PROMs in oncology clinical practice

Of the 132 survey views recorded on Qualtrics, 91(69%) respondents from 56 clinical practices across ANZ agreed to participate (Fig. 1).

Of the 55 respondents who collected PROMs within their clinical practice (*n* = 35), 30 (55%) completed the full survey and an additional 6 (11%) provided partial responses to questions on PROMs collection (in “section 2” of the survey, Additional file 1).

### Respondents characteristics

Respondent characteristics are summarized in Additional file 3: Table S1. Most of the respondents collecting PROMs in clinical practice were from two states in Australia: New South Wales (*n* = 21/55, 38%) and Victoria (*n* = 14/55, 25%). Respondents with the highest response rate were from general referral hospitals (*n* = 20/55), 36%), tertiary referral hospitals (*n* = 15/55, 27%) or cancer centres (oncology units within a tertiary hospital setting focused solely on the treatment of cancer or comprehensive cancer centres; *n* = 14/55, 25%), and from a public setting (*n* = 46/55, 83%). A Chi-square test of independence showed no significant statistical association between collectors of PROMs in clinical practice and non-collectors for state *X*^2^(5, *n* = 73) = 4.25, *p* = 0.51, organisational setting *X*^2^(5, *n* = 73) = 1.44, *p* = 0.92 and organisational type *X*^2^(3, *n* = 73) = 0.49, *p* = 0.92.

### Prevalence of PROM use

**Fig. 2:**
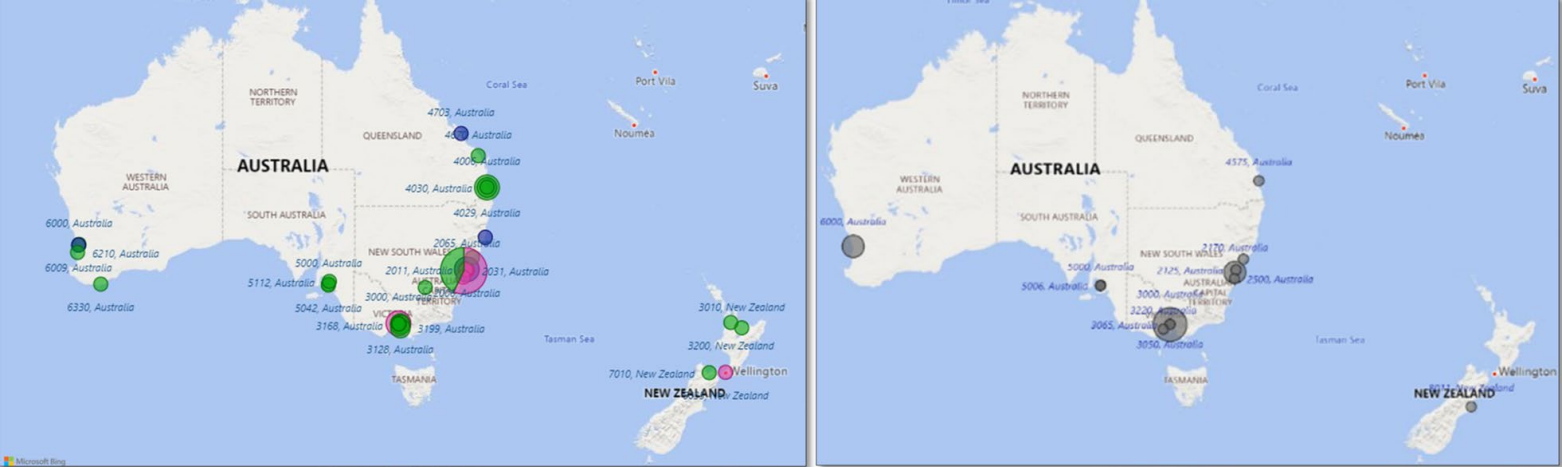
Patterns of PROMs use, and non-use mapped by setting and postcode across ANZ

A geospatial map of postcode data (Fig. 2) shows areas of PROM use across ANZ mapped by the clinical practice setting. The highest response was received from one metropolitan postcode in New South Wales, Australia (*n* = 7) and two or more responses were received from eight other postcodes within New South Wales, Victoria and Queensland, Australia. In comparison, 15 of the 18 respondents who indicated that PROMs were not routinely collected within their clinical practice is highlighted in the insert (Fig. 2). No responses to the survey were received from any clinical practices in the Australian Capital Territory, Northern Territory or Tasmania.

Approximately 80% (*n* = 73/91) of respondents indicated that they collected PROMs. However, 25% (*n* = 18/73) collected PROMs for reasons other than in clinical practice to guide care (Fig. 1). Of the 20% who stated they did not collect PROMs, reasons provided included (1) human resources, lack of integration into electronic medical records and the imbalance between available medical resources and patient numbers (*n* = 3); (2) some practices were in the process of evaluating relevant and flexible PROMs within their clinical practice e.g. supportive care/survivorship or premalignant/benign lesions (*n* = 2); and (3) one respondent felt PROMs were difficult to implement in their setting (paediatrics). Others highlighted the relevance of PROMs to all cancer types and the importance of their integration into routine clinical care (*n* = 2).

### PROMs collection characteristics

Thirty-six of 55 respondents (65%) responded to this section of the survey (Table 1). PROMs were collected in all cancer types (*n* = 21/36, 58%), in adults and older adults (*n* = 15/36, 69%), and in all disease stages (*n* = 31/36, 86%). Many responded that they collected and used PROMs in an outpatient care setting (*n* = 23/36, 64%). Almost half reported collecting PROMs between the last 2–5 years (*n* = 17/36, 47%) and capturing PROMs data in at least half of their patient population (*n* = 17/36, 48%). Almost all (*n* = 33/36, 92%) distributed their PROMs only in English. Seventy five percent (*n* = 27/36) collected PROMs to facilitate communication between provider and patient, improving patient satisfaction with health care, unmet needs, to recognise/screen problems associated with the disease and treatment and to inform management of patient care. Half of the respondents (*n* = 18/36) used generic PROMs plus at least one disease specific, psychological, or customised PROMs developed ‘in house’. The choice of PROMs was often based on the recommendations of others, global standards, or based on the utility of the instrument. PROMs were completed on multiple occasions (*n* = 14/36, 39%) or in association with a consultation (*n* = 12/36, 33%) without an overlap in responses. Approximately 40% (*n* = 14/36) of respondents used various modes of administration to collect PROMs data and over 60% (*n* = 22/36) stored their data with their hospital system either within the electronic medical records or a purpose-built system.

**Table 1:** PROMs Collection Characteristics

PROMs collection	*n* = 36 (%)
**Type of cancers**	
All cancer types	21 (58)
Lung	4 11)
Melanoma	2 (6)
Prostate	3 (8)
Multiple	2 (6)
Other*	4 (11)
**Population**	
Adults only	12 (33)
Adults, older adults	13 (36)
Adolescents and young adults, adults, older adults	8 (22)
Other^#^	3 (8)
**Disease stage**	
All stages	31 (86)
Early stage	3 (8)
Other (metastatic, survivorship)	2 (6)
**Clinical setting**	
Acute hospital inpatient care	2 (6)
Acute hospital inpatient care and Outpatient care	8 (22)
Outpatient care	23 (64)
Other^^^	3 (8)
**Professional discipline**	
Allied health	3 (8)
Medical oncology	3 (8)
Medical oncology, nursing	3 (8)
Medical and radiation oncology	3 (8)
Medical oncology, radiation oncology, nursing	3 (8)
Medical and radiation oncology, nursing, allied health	2 (6)
Nursing	9 (25)
Radiation oncology	2 (6)
Not discipline specific	3 (8)
Other**	5 (14)
**Number of years for PROMs use**	
< 2	5 (14)
2—5	17 (47)
6–10	6 (17)
> 10	4 (11)
Do not know	4 (11)
**% of Patients with PROMs data collected/year**	
< 25%	8 (22)
25–50%	5 (14)
50–75%	6 (17)
> 75%	11 (31)
Do not know	6 (17)
**Language**	
English	33 (92)
English, other (Chinese)	3 (8)
**Reason(s) for PROMs collection**	
Facilitate communication between provider and patient + various other^##^	27 (75)
Screen for mental health issues + various other^##^	5 (14)
Various other^##^	4 (11)
**Type of PROM used**	
Generic	4 (11)
Generic + Other*^#^	18 (50)
Disease/condition specific	3 (8)
Disease/condition specific + Other*^#^	4 (11)
Psychological	2 (6)
Psychological + Other*^#^	2 (6)
Tool developed ‘in house’/within the clinical practice	2 (6)
Not specified	1 (3)
**Choice of PROMs informed by**	
Based on global standards only	3 (8)
Based on global standards + Other*^^^	7 (19)
On recommendations of others + Other*^^^	8 (22)
Practical/clinical utility of the instrument only	3 (8)
Practical/clinical utility of the instrument + Other*^^^	7 (19)
Reliability and validity of the instrument based on published research	2 (6)
Do not know	2 (6)
Other*^^^	4 (11)
**How often is PROMs completed by a patient**	
At a single-time point	6 (17)
At multiple occasions other than daily	14 (39)
Relative to patient visit/clinical consult/clinician decision	12 (33)
Not specified	4 (11)
**Mode of administration**	
Administered over the telephone + hard copy	4 (11)
Administered over the telephone ± other^^^^	3 (8)
Hard copy given to the patient in clinic or practice only	4 (11)
Electronic in clinic or practice (completed on computer/tablet/smartphone)	2 (6)
Electronic with link sent via email + various other^^^^	14 (39)
Other^^^^	5 (14)
Not specified	4 (11)
**Storage of PROMs data**	
On a purpose-built data collection platform e.g. REDCaP	7 (19)
Within the hospital system (e.g. via electronic medical records)	14 (39)
Within the hospital system (e.g. via electronic medical records) or on a purpose-built data collection platform e.g. REDCaP	8 (22)
Other (secure filing cabinet, clinical database)	3 (8)
Not specified	4 (11)

*Other = Breast, Gastrointestinal, Gynaecology, Supportive care

^#^Other = [Pediatrics, Adults (18 years+), Older Adults (65 years+)], [Adolescents (12–19 years) and Young Adults (20–24 years), Adults], [Adolescents and Young Adults]

^^^Other = Private clinic, Inpatient palliative care unit, Telehealth

**Other = [Medical oncology, Nursing, Allied health ± Haematology], [Surgery ± nursing], [Treatment decision]

^##^Various Other = Improve patient satisfaction with health care, detect unmet needs, Recognise/screen problems (e.g., symptoms/side effects) associated with the disease and treatment, Feedback on symptoms or side-effects to clinicians, Inform management, predict prognosis

*^#^Other = Disease/Condition specific, Psychological, Patient Reported Experience Measures, Tool developed ‘in house’

*^^^Other = Availability and cost of the instrument, based on global standards), Practical/clinical utility of the instrument, Reliability and validity of the instrument based on published research,,Length of time required to complete the instrument

^^^^Other = Administered over the telephone, administered via video call,SMS with link sent via text message on mobile phone, Hard copy sent via post, Electronic in clinic or practice (completed on computer/tablet/smartphone)

### PROMs type and reason for PROMs collection by clinical setting

The type of PROMs used by the different clinical settings and how the results were used are summarized in Additional file 3: Table S2. Generic PROMs in combination with various other (*n* = 7/20, 35%) were the most used in general referral hospitals, to facilitate communication and for many other reasons (*n* = 8/20, 40%). Similarly, generic PROMs in combination with various other (*n* = 6/15, 40%) were the most used in tertiary hospitals and the main reasons for collection included facilitating communication and various other (*n* = 7/15, 47%), screen for mental health issues and various other (*n* = 2/15, 13%) and improve patient satisfaction, recognise and screen problems plus various other, and detect unmet needs plus various other. Generic PROMs in combination with various other (*n* = 6/9, 67%) were mainly used in cancer centres to facilitate communication and for various other reasons (*n* = 6/9, 67%). In other settings, psychological in combination with other (*n* = 2/6, 33%) and disease / condition specific PROMs (*n* = 2/6, 33%) were used to facilitate communication with other reasons (*n* = 3/6, 50%) and to screen for mental health issues (*n* = 2/6, 33%).

### Resourcing and reporting

Thirty respondents (55%) provided responses for this section (Table 2). Support for the collection of PROMs was provided via organizational funding (*n* = 9/30, 30%) or from within the individual clinical practice (*n* = 9/30, 30%). Oversight on PROMs collection and reporting was provided by a steering committee in 30% (*n* = 9/30) of cases and around a quarter (*n* = 7/30) did not know who provided governance. More than half (*n* = 17/30) did not have a database coordinator to manage PROMs collection. Approximately two-thirds (*n* = 20/30, 67%) of the respondents reported that PROM data was made available to clinicians in real-time. Doctors, nurses and allied health professionals were the most common disciplines with access to the PROMs data and data was reported to various key stakeholders from individual clinicians to health services and others such as funders and industry, government departments, and in peer reviewed publications. Over half (*n* = 16/30, 53%) did not know how often their clinical practice reported on PROMs data or to other stakeholders (e.g. individual clinicians or patients).

**Table 2:** Resourcing and Reporting

Resourcing and reporting	*n* = 30 (%)
**Funding**	
Grant funding	4 (13)
Organisational funding	9 (30)
The individual specialty/clinical practice	9 (30)
Do not know	6 (20)
Other (philanthropic organisations, existing resources)	2 (7)
**Oversight is provided by**	
Clinical governance unit within the organisation	2 (7)
Clinical governance unit within the organisation, patients e.g. consumer advocates or groups	2 (7)
Steering Committee or working group within the clinical practice/unit	9 (30)
Steering Committee or working group within the clinical practice/unit, clinical governance unit within the organisation	3 (10)
Steering Committee Or working group within the clinical practice/unit + various others*	3 (10)
Do not know	7 (23)
Other (patients, clinicians, or no oversight provided)	4 (13)
**Coordinator/database manager managing the collection and use of PROMs**	
Yes	8 (27)
No	17 (57)
Do not know	5 (17)
**PROM availability to clinicians in real-time to support clinical care**	
Yes	20 (67)
No	8 (27)
Do not know	2 (7)
**Disciplines with access to PROMs and ability to use the information provided by it**	
Doctors, nurses, allied health	13 (43)
Doctors, nurses, allied health and other (data manager, aboriginal health workers and aboriginal liaison officers)	3 (10)
Doctors, Nurses	2 (7)
Doctors, nurses, allied health, patients and consumers	2 (7)
Allied health	2 (7)
Nurses	2 (7)
Do not know	1 (3)
Other (external staff e.g. survivorship team, various other)	5 (17)
**Reporting to key stakeholders**	
Health services	2 (7)
Health services and various other^#^	4 (13)
Individual clinicians	6 (20)
Individual clinicians, Health services	3 (10)
Individual clinicians, Peer-reviewed publications and journals, conferences and forums	2 (7)
Individual clinicians and various other^*#^	7 (23)
No one	2 (7)
Do not know	3 (10)
Other (patients and consumers, funders and industry)	1 (3)
**How is PROMs reported?**	
At individual level e.g., scores and changes in response	9 (30)
In aggregated form (e.g. annual reports, peer-reviewed publications)	2 (7)
At individual level and in aggregated form	3 (10)
At individual level, In aggregated form, and used for comparisons (e.g. patient results compared with cohort)	5 (17)
Does not report	3 (10)
Do not know	4 (13)
Various other**	4 (13)
**Reporting frequency to key stakeholders**	
Annually	4 (13)
Monthly	2 (7)
Do not know	16 (53)
Other^##^	8 (27)

*Various other = Clinical governance unit within the organisation, Funders e.g., industry, Patients e.g., consumer advocates or groups

^#^Various other = [Funders and industry, Peer-reviewed publications and journals, conferences and forums], [Government departments], [Patients and consumers, Peer-reviewed publications and journals, Conferences and forums]

*^#^Various other = Health services, Funders and industry, Government departments, Peer-reviewed publications and journals, Conferences and forums

**Various other = [At individual level, does not report], [In aggregated form, Used for comparisons], [At individual level, Used for comparisons], other

^##^Other = Not reported, following completion by patient, daily, as per project timelines, after initial/final consultation

### Impact of PROMs in clinical practice

Of the thirty respondents who completed the full survey, 19 (63%) stated that the collection and reporting of PROMs had a positive impact on patient outcomes including “*increased allied health referrals and reduced presentations to acute assessment unit in oncology*”; “*positive feedback from consumers and demonstrated reduced emergency department presentations and admissions*”; “*concerns were identified early in their diagnosis and again if there are any changes*”; that there was “*increased clinical accountability*”; scores above a certain threshold allowed input from psychology or a social work; and the positive changes were noted “*according to the annual report*”. Seven of the 30 respondents (23%) were not aware of any positive impacts whilst 4 (13%) were unsure or were “*unable to determine until some analysis*” could be undertaken.

## Discussion

This study provides an insight into the current landscape of PROMs use in oncology care across ANZ. The majority of the respondents were from NSW, hospital, outpatient care and a public setting. PROMs were collected in all cancer types, in all stages of the disease, in an adult population, applied in English, and used to facilitate communication with other reasons that included improving patient satisfaction, detecting unmet needs, recognising and screening problems and providing feedback on symptoms or side-effects to clinicians. This study also highlights, the limited resources for PROMs implementation, a lack of systematic priority driven approach, and the variation in PROMs uptake between the two countries and in certain jurisdictions within Australia.

The percentage of patients with PROMs data collected, how the choice of PROMs was informed, timing, frequency, mode of collection and storage of data varied substantially. These findings were similar to a study conducted in the United States (US) by Zhang and colleagues who observed variability in timing and frequency with several practices experimenting with their own PRO collection timelines and highlight the need for greater standardisation of PRO use [13]. English was the most common language in which PROMs were distributed which may inadvertently exclude individuals with limited English proficiency in the PROMs informed clinical decision-making process raising concerns about equity of access to this clinical tool. Institutional leadership and funding support for validating translations and adoption of PROMs in other languages is needed. Multi-institutional collaboration with hospitals that serve larger non-speaking populations may decrease the resource burden and costs for adapting and implementing PROMs in non-English speaking populations [15].

A third of responders who completed the last section reported that PROMs data collection was resourced by the individual clinical practice itself and approximately a quarter did not know who provided oversight over PROMs data collection with over half reporting the lack of a coordinator or database manager. A coordinator is an important facilitator to help overcome the labour-intensive nature of implementation and barriers such as time constraints and an absence or lack of staff coordination. In addition, the coordinator acts as a point of contact, provides a link between the different levels within the organisation, and supports the multidisciplinary team [16].

Reporting of PRO data was delivered to a broad set of stakeholders in various ways e.g., at individual level using scores and change in response or in aggregate form for annual reports but reporting frequency was unknown to over half of the respondents again highlighting the need for standardisation as well as explicit governance of PROs. Further, in comparison to those that collected PROMs within their clinical practice, 20% (*n* = 18/91) of initial respondents in this study stated they did not collect PROMs. Some cited that this was due to barriers that included lack of organisational resources and integration. Other studies have also highlighted major barriers for health professionals that include lack of knowledge on interpretation and integration of PROMs into their clinical practice and inability with electronic PRO systems. Prevalent service level barriers are integration into clinical workflows and inadequate information technology infrastructure to enable ease of PRO data collection [17]. To address such barriers a US study implemented PROMs in cancer care that included real-time reporting, and effective resourcing and governance using an integrated health system approach [18]. The program used iterative cycles of implementation and review which involved engagement of multidisciplinary providers representing a wide breadth of clinical disease sites, strong support from cancer leadership focused on the implementation domain rather than disease specific instruments, having a physician champion, local supervisors and integrated case managers for each clinical unit [18]. Similar approaches could be adopted in Australia and New Zealand. Challenges with technology, clinician uncertainty as to how to use PROMs and competing priorities impacting integration of these measures into workflows remain consistent barriers [19, 20]. Important organisational considerations are the readiness to implement PROMs, addressing the current capabilities, existing resources and infrastructure; addressing barriers, by articulating a clear pathway for overcoming barriers; developing implementation strategies aimed to identify and support the PRO champions, integrating and piloting collection of PROs and consideration of an implementation framework; monitoring use and evaluating outcomes, and reporting data back to key stakeholders; and sustainability, that includes the development of a protocol for PROM collection and providing regular training [21].

This study has notable limitations. Although the developed questionnaire was reviewed by the COSA working group, the reliability of the questionnaire was not assessed using any statistical method such as the test–retest approach. Further, approximately 30% (*n* = 41/132) non-responders viewed the survey but did not agree to participate. As consent to participate was requested upfront, the characteristics of the non-responders were not captured in our survey to allow for comparison between the responders and non-responders to assess non-response bias. In addition, although our survey drew responses from 56 clinical practices across ANZ it is difficult to ascertain the generalizability and representativeness of our results. The respondents to our survey represent a sample of the oncology clinical practices across ANZ and may not be representative of all other practices, especially those who do not collect PROMs data and may be less inclined to complete our survey. There were a higher number of responders (*n* = 86) from Australia in comparison to New Zealand (*n* = 5). However, this may be expected given the larger Australian population, geography and associated number of clinical practices in comparison to New Zealand. There were also a large number of respondents from New South Wales and Victoria, Australia, and similarly these results may be due to these being the two largest regions in Australia by population. We received no responses from any clinical practices in the Australian Capital Territory, Northern Territory, or Tasmania suggesting less uptake of PROMs in these states and territories with lower population density and more limited healthcare resources. Further research should examine whether there is a lack of knowledge or data on the potential benefits of PROMs in routine clinical practice, or a lack of infrastructure to support PROMs uptake in certain jurisdictions [20].

Australia and New Zealand have large indigenous populations who may benefit from the implementation of culturally specific PROMs and yet our survey demonstrated little evidence of PROMS specifically used in Indigenous populations although some work is underway to develop a wellbeing measure for Aboriginal and Torres Strait Islander adults [22]. Further research is required to address the implementation of PROMS for these populations.

### Implications for practice and research

This research has important implications highlighting the need for a systematic approach to PRO use including minimum standards of PRO use, standardised PROMs and implementation strategies that ensure equity of access [9]. Future research should focus on unique PRO needs of specific population such as Indigenous, CALD and those from rural and remote populations where access to technology supporting PROMS may be more limited. Implementation research focusing on the best implementation strategies to facilitate adoption, scale up and sustainability would also be of value.

## Conclusion

PROM use across Australia and New Zealand seems variable and occurring predominantly in larger metropolitan centres with limited standardisation of approach and implementation. Considering the significant benefits of PROMs use, greater focus on equitable adoption of PROMs in diverse cancer care settings is urgently needed.

### Supplementary Information

**Additional file 1: Appendix 1.** PROMs Survey.

**Additional file 1: Appendix 2.** Social Media Campaign.

**Additional file 3.** **Suppl Table 1**, Respondent Characteristics; and **Suppl Table 2**, Type of PROMs and Reason for PROMs Collection by Clinical Setting.

## Data Availability

All data generated or analysed during this study are included in this published article [and its supplementary information files].

## Declarations

**Ethical Approval**

This study was approved by the Monash University Human Research Ethics Committee (MUHREC #29941). A research collaboration agreement was drafted and signed between Monash University, COSA and ISOQOL to conduct this study.
