# Supplementary material for: The use of patient reported outcome measures in oncology clinical practice across Australia and New Zealand
Source: J Patient Rep Outcomes. 2024 Jan 2;8:1. doi: 10.1186/s41687-023-00664-x (PMC10761654; doi:10.1186/s41687-023-00664-x)
Supplement: Supplementary file 1 — Additional file 1: Appendix 1. PROMs Survey. [file 41687_2023_664_MOESM1_ESM.docx]

Understanding Patient Reported Outcome Measures (PROMs) Use in Oncology Clinical Practice across ANZ

Start of Block: INTRODUCTION

**UNDERSTANDING PATIENT REPORTED OUTCOME MEASURES USE IN ONCOLOGY CLINICAL PRACTICE**

**PURPOSE OF THIS SURVEY:**You are invited to take part in this study to examine and provide an understanding of how patient reported outcome measures (PROMs) are used in clinical practice with patients diagnosed with cancer across Australia and New Zealand.

PROMs are questionnaires that directly capture patient’s perceptions. For example, they might ask patients to report on their disease or treatment related symptoms or side-effects, mental health concerns, or unmet needs. In your clinical practice you may be using PROMs to screen mental health and functional impacts such as anxiety and/or depression; for clinical monitoring of the impact of treatment side effects; or to facilitate patient-provider communication. 

The purpose of this study is to describe how oncology clinical practices are using PROMs for the management of individual ***patients with cancer***. When we refer to clinical practice we mean any hospitals, outpatient specialty clinics and community services.

This research has been reviewed by the Monash University Human Research Ethics Committee (Project ID 29941) and is supported by the International Society of Quality of life (ISOQOL) and the Clinical Oncology Society Australia (COSA). Further information on this survey can be downloaded here: [Participant information sheet](https://monash.az1.qualtrics.com/CP/File.php?F=F_3VpSdsoGSXLGVee)

**HOW TO COMPLETE THIS SURVEY?**  This short online survey comprises a maximum of 34 questions. You may wish to complete this questionnaire because you manage patients with cancer, and are a doctor, nurse or allied health professional or are a representative who has a good understanding of the rationale for the inclusion of PROMs in their clinical practice or setting and how they are collected and used. If you believe that you do not have the knowledge or experience to make an informed decision on any particular question, you can just leave the question blank. The survey is expected to take approximately 10 minutes to complete. ***Please note:*** For ***Q1.6***, if *'clinical practice to guide patient care*' is not selected as an option, then the survey will end. Further, PROMs maybe used in different areas of an organisation to guide cancer care. We are very interested to capture these different pockets of activity and perspectives. Please forward this survey within your organisation to other areas managing patients with cancer.

**TO SAVE AND CONTINUE LATER:** You can save the survey and return to it later by simply closing the survey.  Qualtrics automatically saves your progress using cookies.  However, please note this will only work as long as you return to the survey on the same internet browser and on the same computer.  Completion of the online survey is due by ***Sunday 1 May, 2022***.

**CONSENT:**Your participation in this research study is voluntary and you can withdraw at any time. Your responses will be non-identifiable and we do not collect identifying information such as your name, email address or IP address. All data is stored in a password protected electronic format. If you have any questions about this study, please contact *ashika.maharaj@monash.edu*

0.0 Clicking on the "***agree***" button below indicates that:
• you have read the above information
• you voluntarily agree to participate
• you are at least 18 years of age
 
If you do not wish to participate in the research study, please decline participation by clicking on the "***disagree***" button.

- agree (1)
- disagree (2)

Skip To: End of Survey If Clicking on the "agree" button below indicates that:• you have ready the above information• you v... = disagree

End of Block: INTRODUCTION

Start of Block: 1. CLINICAL PRACTICE RELATED INFORMATION

1.1 Name of clinical practice or unit and/or organisation

________________________________________________________________

| 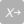 |
| --- |

1.2 Please select the country of your clinical practice / unit / organisation

- Australia (1)
- New Zealand (2)

1.3 Please enter your postcode/region of clinical practice / unit / organisation

________________________________________________________________

| 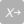 |
| --- |

1.4 Does your clinical practice use or collect PROMs? This may include questionnaires (tools) such as the distress thermometer, or tools to screen for supportive care needs. Consider tools that you may use for screening e.g. the hospital anxiety and depression scale, identification of issues e.g. brief pain inventory for pain related issues or understanding patient risks. *(****Please note:*** *We are interested in the experience at your clinical practice regardless of whether you yourself use PROMs; please talk to your colleagues before, if you are thinking of answering NO)*

- Yes (1)
- No (2)

Skip To: 1.6 If Does your clinical practice use or collect PROMs? This may include questionnaires (tools) such as... = Yes

Display This Question:

If Does your clinical practice use or collect PROMs? This may include questionnaires (tools) such as... = No

| 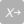 |
| --- |

1.5a If no, does your clinical practice intend to collect PROMs for individual patient care in the near future?

- Yes (1)
- No (2)

Display This Question:

If Does your clinical practice use or collect PROMs? This may include questionnaires (tools) such as... = No

1.5b Are there any further comments or suggestions?

________________________________________________________________

Skip To: End of Survey If Condition: Are there any further comme... Is Empty. Skip To: End of Survey.

Skip To: End of Survey If Condition: Are there any further comme... Is Not Empty. Skip To: End of Survey.

1.6 At my clinical practice / unit / organisation, PROMs are collected (select all that apply)

- In clinical practice to guide individual patient care (1)
- Within a research / clinical trial (2)
- For quality improvement purposes (3)
- For a clinical quality registry (4)
- Other (5) ________________________________________________

End of Block: 1. CLINICAL PRACTICE RELATED INFORMATION

Start of Block: 2. PROM COLLECTION

| 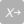 |
| --- |

2.1 In which of the following cancer(s) does your clinical practice use PROMs for individual patient care? (*select all that apply*)

- All cancer types (1)
- Bladder (2)
- Breast (3)
- Gynaecology (4)
- Gastrointestinal (5)
- Head and Neck (6)
- Haematological (7)
- Kidney (8)
- Lung (9)
- Melanoma (-1)
- Palliative Oncology (10)
- Prostate (11)
- Other (*please specify*): (12) ________________________________________________

2.2 In which population does your clinical practice collect PROMs for individual patient care? (*select all that apply*)

- Pediatrics (1)
- Adolescents and Young Adults (2)
- Adults (3)
- Older Adults (4)

2.3 At what stage of the disease are PROMs collected and used? (*select all that apply*)

- All stages (1)
- Early stage (2)
- Locally advanced (3)
- Metastatic (4)
- Other (*please specify*): (5) ________________________________________________

2.4 In which clinical setting is the PROMs collected and used? (*select all that apply*)

- Acute hospital inpatient care (1)
- Outpatient care (2)
- Community care (3)
- Other (*please specify*): (4) ________________________________________________

2.5 In which professional discipline is the PROM collected and used? (select all that apply)

- Medical oncology (1)
- Surgery (2)
- Radiation oncology (3)
- Nursing (4)
- Allied health (5)
- Community care (6)
- Not discipline specific (7)
- Other (*please specify*): (8) ________________________________________________

2.6 Approximately how many years has the clinical practice been using PROMs?

- < 2 years (1)
- 2 - 5 years (2)
- 6 - 10 years (3)
- > 10 years (4)
- Do not know (5)

2.7 Approximately, what percentage of patients per year will have their PROMs collected within your clinical practice and used for patient care

- < 25% (1)
- 25 - 50% (2)
- 50 - 75% (3)
- > 75% (4)
- Do not know (5)

2.8 In which language is the PROMs collected and used? (*select all that apply*)

- English (1)
- Chinese (2)
- Arabic (3)
- Italian (4)
- Vietnamese (5)
- Greek (6)
- Other (*please specify*): (7) ________________________________________________

2.9 What is the reason for collecting PROMs? (*select all that apply*)

- Facilitate communication between provider and patient (1)
- Improve patient satisfaction with health care (2)
- Screen for mental health issues (3)
- Detect unmet needs (4)
- Recognise/screen problems (e.g. symptoms/side effects) associated with the disease and treatment (5)
- Feedback on symptoms or side-effects to clinicians (6)
- Inform management (7)
- Predict prognosis (8)
- Other (*please specify*): (9) ________________________________________________

2.10 What type of PROM does your clinical practice use? (*select all that apply*)

- Generic (tools that capture the health status and quality of life common to most patients/general population e.g. The EQ-5D questionnaire) (1)
- Disease/condition specific (2)
- Psychological (3)
- Patient reported experience measures (4)
- Tool developed ‘in house’ / within the clinical practice (5)
- Other (*please specify*): (6) ________________________________________________

2.11 Please list all the PROMs that your clinical practice uses for collecting PRO data

________________________________________________________________

2.12 Which of the following informed the choice of PROMs within your clinical practice? (*select all that apply*)

- On recommendations of others (10)
- Based on global standards (i.e. International Consortium for Health Outcomes Measurement) (12)
- Practical/clinical utility of the instrument agree (13)
- Availability and cost of the instrument (14)
- Presentation/layout of the instrument (11)
- Reliability and validity of the instrument based on published research (15)
- Length of time required to complete the instrument (16)
- Developed a new instrument for our purposes [if yes, please answer question 2.13] (17)
- Other (*please specify*): (18) ________________________________________________

Display This Question:

If Which of the following informed the choice of PROMs within your clinical practice? (select all th... = Developed a new instrument for our purposes [if yes, please answer question 2.13]

2.13 If your clinical practice developed a new instrument, what was the development process and were patients involved?

________________________________________________________________

2.14 How often does a patient at your clinical practice complete a PROM?

- Relative to patient visit/clinical consult/clinician decision (1)
- At a single-time point (*please specify e.g. once a year*) (2) ________________________________________________
- At multiple occasions other than daily (3)

2.15 Please specify the mode of administration for PRO/PROM collection (*select all that apply*)

- Electronic with link sent via email (1)
- Administered over the telephone (2)
- Administered via video call (3)
- SMS with link sent via text message on mobile phone (4)
- Hard copy sent via post (5)
- Electronic in clinic or practice (completed on computer/tablet/smartphone) (6)
- Hard copy given to the patient in clinic or practice (7)
- Other (*please specify*): (8) ________________________________________________

2.16 Where is the data that is collected stored within your clinical practice?

- Within the hospital system (e.g. via electronic medical records) (1)
- On a purpose-built data collection platform e.g. REDCaP (2)
- In a secure locked filing cabinet (4)
- Other (*please specify*): (3) ________________________________________________

End of Block: 2. PROM COLLECTION

Start of Block: 3. RESOURCING, REPORTING, AND IMPACT OF PROMS IN CLINICAL PRACTICE

| 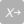 |
| --- |

3.1 The collection of PROMs within your clinical practice (may include the tools, staff, infrastructure etc) is primarily funded by:

- The individual specialty / clinical practice (1)
- Organisational funding (2)
- Grant funding (3)
- Consumer groups e.g. the Liver Foundation (4)
- Philanthropic organisations (5)
- Do not know (7)
- Other (*please specify*): (6) ________________________________________________

3.2 Oversight for PROMs collection within your clinical practice is provided by (*select all that apply*):

- Steering Committee or working group within the clinical practice / unit (1)
- Clinical governance unit within the organisation (2)
- Funders e.g. industry (3)
- Patients e.g. consumer advocates or groups (4)
- Do not know (6)
- Other (*please specify*): (5) ________________________________________________

3.3 Does your clinical practice have a coordinator/database manager who manages the collection and use of PROMs?

- Yes (1)
- No (2)
- Do not know (4)

3.4 Has the PROM been made available to clinicians in real-time to support clinical care?

- Yes (*please specify*)*:* (1) ________________________________________________
- No (2)
- Do not know (4)

3.5 Who in your practice/unit/service has access to the PROM and is able to use the information provided by it? (*select all that apply*)

- Doctors (1)
- Nurses (2)
- Allied Health (e.g. pharmacists, occupational therapists, physiotherapists) (3)
- Patients and consumers (4)
- Do not know (6)
- Other (*please specify*): (5) ________________________________________________

3.6 To whom (key stakeholders) does your clinical practice report the collected PROM? (*select all that apply*)

- Individual clinicians (1)
- Health services (2)
- Patients and consumers (3)
- Funders and industry (4)
- Government departments (5)
- Peer-reviewed publications and journals (6)
- Conferences and forums (7)
- No one (8)
- Do not know (10)
- Other (*please specify*): (9) ________________________________________________

3.7 How does your clinical practice report on PROMs? (*select all that apply*)

- At individual level e.g., scores and changes in response (1)
- In aggregated form (e.g. annual reports, peer-reviewed publications) (2)
- Used for comparisons (e.g. patient results compared with cohort) (3)
- Does not report (4)
- Do not know (6)
- Other (*please specify*): (5) ________________________________________________

3.8 How often does your clinical practice report on PROMs?

- Daily (1)
- Monthly (2)
- Annually (3)
- Do not know (5)
- Other (*please specify*): (4) ________________________________________________

3.9 Are you aware if the collection and reporting of PROMs has had a positive impact on patient outcomes?

________________________________________________________________

3.10 Can you think of anything else that you would like to add about the collection and use of PROMs at your clinical practice/service?

________________________________________________________________

End of Block: 3. RESOURCING, REPORTING, AND IMPACT OF PROMS IN CLINICAL PRACTICE

Start of Block: 4. FUTURE RESEARCH

4.1 Would you be interested in participating in a future qualitative study and/or community of practice related to PROMs?

- Yes (1)
- No (2)

Skip To: End of Survey If Would you be interested in participating in a future qualitative study and/or community of practi... = No

End of Block: 4. FUTURE RESEARCH
