# Supplementary figures and images for: The use of patient reported outcome measures in oncology clinical practice across Australia and New Zealand
Source: J Patient Rep Outcomes. 2024 Jan 2;8:1. doi: 10.1186/s41687-023-00664-x (PMC10761654; doi:10.1186/s41687-023-00664-x)

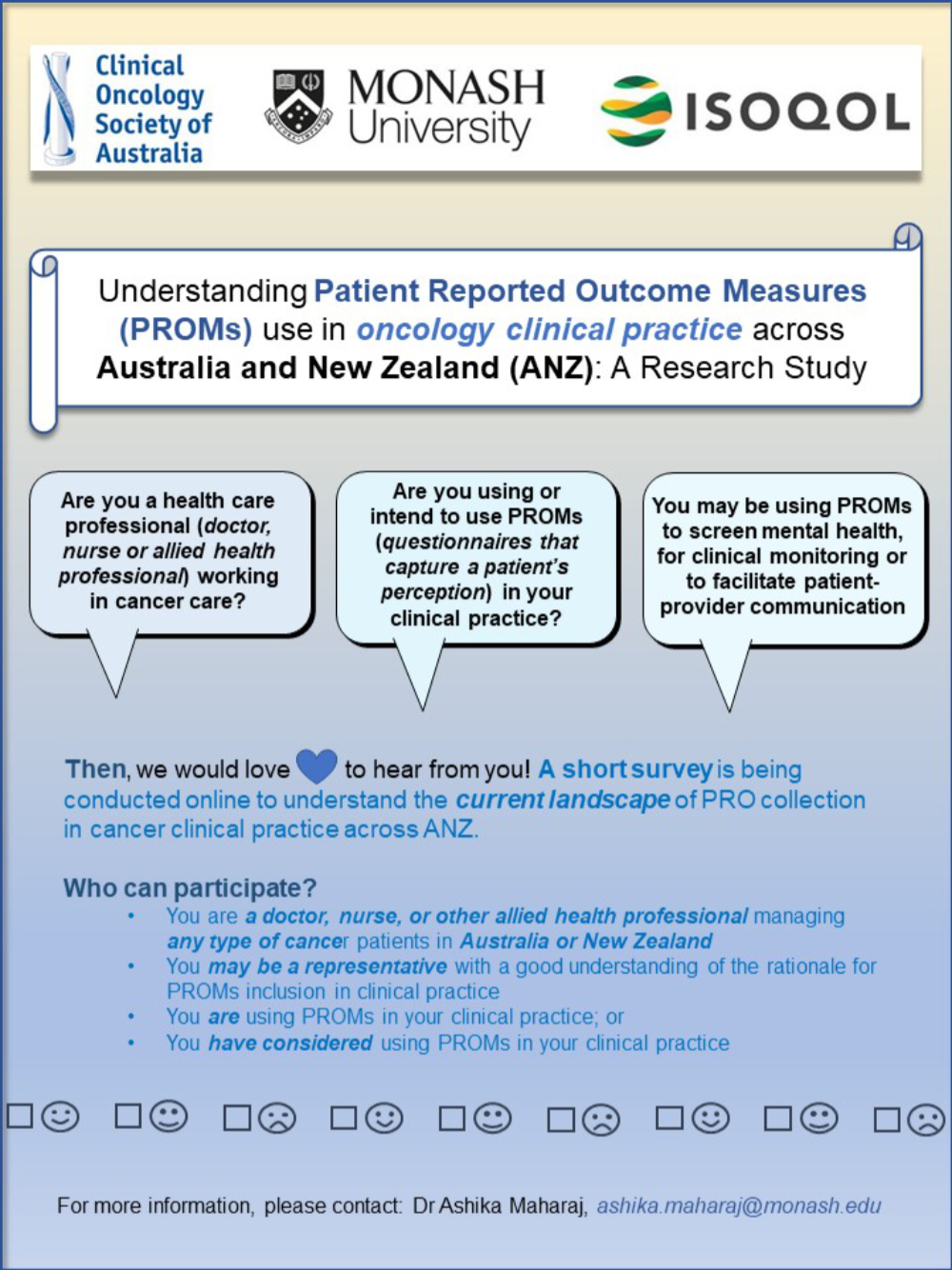

Supplement: Supplementary file 2 — Additional file 1: Appendix 2. Social Media Campaign. [file 41687_2023_664_MOESM2_ESM.jpg]
