# Supplementary material for: The use of patient reported outcome measures in oncology clinical practice across Australia and New Zealand
Source: J Patient Rep Outcomes. 2024 Jan 2;8:1. doi: 10.1186/s41687-023-00664-x (PMC10761654; doi:10.1186/s41687-023-00664-x)
Supplement: Supplementary file 3 — Additional file 3. Suppl Table 1, Respondent Characteristics; and Suppl Table 2, Type of PROMs and Reason for PROMs Collection by Clinical Setting. [file 41687_2023_664_MOESM3_ESM.docx]

Supplementary Table 1: Respondent Characteristics

| **RESPONDENT CHARACTERISTICS** | | ***Overall n=91*** (%) | | ***PROMs in CP***  ***n=55 (%)*** | |
| --- | --- | --- | --- | --- | --- |
| REGION | New South Wales, Australia | 28 | (31) | 21 | (38) |
|  | Queensland, Australia | 14 | (15) | 9 | (16) |
|  | South Australia | 8 | (9) | 3 | (5) |
|  | Victoria, Australia | 27 | (30) | 14 | (25) |
|  | Western Australia | 8 | (9) | 4 | (7) |
|  | New Zealand | 5 | (5) | 4 | (7) |
|  | Undisclosed | 1 | (2) | - | - |
| SETTING | Cancer Centre^*^ | 12 | (13) | 9 | (16) |
|  | Comprehensive Cancer Centre | 8 | (9) | 5 | (9) |
|  | General/Referral Hospital | 27 | (30) | 20 | (36) |
|  | Tertiary Hospital | 29 | (32) | 15 | (27) |
|  | University | 5 | (5) | - | - |
|  | Other^#^ | 10 | (11) | 6 | (11) |
| ORGANISATION TYPE | Public | 76 | (84) | 46 | (83) |
|  | Private | 13 | (14) | 8 | (15) |
|  | Other^**^ | 2 | (2) | 1 | (2) |

**Cancer centre =** Clinical practices within tertiary hospitals focused solely on the treatment of cancer (NB: responders that referred to their clinical practice as cancer services are not included here)

**Comprehensive cancer centre** = integrated cancer research, treatment and education centre

**Other^#^** = Cancer Foundation; Medical Imaging; Oncology Network; State Institute; Support Group; Undisclosed

**Other^**^** = Non-Governmental Organisation; Undisclosed

Supplementary Table 2: Type of PROMs and Reason for PROMs Collection by Clinical Setting

*NB: n=1 if not stated*

| **Clinical Setting** | **Type of PROM Used** | **Reason for PROMs collection** |
| --- | --- | --- |
| **General referral hospital *(n=20)*** | Disease/condition specific | Various Other^##^ |
|  | Generic *(n=2)* | Facilitate communication between provider and patient + Various Other^##^ *(n=2)* |
|  | Generic + Other^*#^ *(n=5)* | Facilitate communication between provider and patient + Various Other^##^ *(n=3)* |
|  |  | Screen for mental health issues + Various Other^##^ |
|  |  | Not specified |
|  | Other *(n=2)* | Facilitate communication between provider and patient + Various Other^##^ *(n=2)* |
|  | Tool developed ‘in house’ / within the clinical practice | Facilitate communication between provider and patient + Various Other^##^ |
|  | Not specified *(n=9)* | ─ |
| **Tertiary hospital *(n=15)*** | Disease/condition specific | Various Other^##^ |
|  | Disease/condition specific + Other^*#^ *(n=2)* | Facilitate communication between provider and patient + Various Other^##^ *(n=2)* |
|  | Generic | Facilitate communication between provider and patient + Various Other^##^ |
|  | Generic + Other^*#^ *(n=5)* | Various Other## *(n=2)* |
|  |  | Facilitate communication between provider and patient + Various Other^##^ *(n=2)* |
|  |  | Screen for mental health issues + Various Other^##^ |
|  | Psychological | Facilitate communication between provider and patient + Various Other^##^ |
|  | Psychological + Other*^#^ | Facilitate communication between provider and patient |
|  | Tool developed ‘in house’ / within the clinical practice | Screen for mental health issues + Various Other^##^ |
|  | Not specified *(n=3)* | ─ |
| **Cancer Centre *(n=9)*** | Generic | Facilitate communication between provider and patient + Various Other^##^ |
|  | Generic + Other^*#^ *(n=5)* | Facilitate communication between provider and patient + Various Other^##^ *(n=5)* |
|  | Not specified *(n=3)* | ─ |
| **Other^#^ *(n=6)*** | Disease/condition specific + Other^*#^ *(n=2*) | Facilitate communication between provider and patient + Various Other^##^ *(n=2)* |
|  | Generic + Other^*#^ | Facilitate communication between provider and patient + Various Other^##^ |
|  | Psychological | Screen for mental health issues + Various Other^##^ |
|  | Psychological + Other^*#^ | Screen for mental health issues + Various Other^##^ |
|  | Not specified | ─ |
| **Comprehensive Cancer Centre *(n=5)*** | Disease/condition specific + Other^*#^ | Facilitate communication between provider and patient + Various Other^##^ |
|  | Generic + Other^*#^ | Facilitate communication between provider and patient + Various Other^##^ |
|  | Not specified *(n=3)* | ─ |

^#^ Other = Cancer Foundation; Medical Imaging; Oncology Network; State Institute; Support Group; Undisclosed

*# Other = Disease/Condition specific, Psychological, Patient Reported Experience Measures, Tool developed ‘in house’

^##^ Various Other = Improve patient satisfaction with health care, detect unmet needs, Recognise/screen problems (e.g., symptoms/side effects) associated with the disease and treatment, Feedback on symptoms or side-effects to clinicians, Inform management, predict prognosis
